# Identification of prognostic signature of non–small cell lung cancer based on TCGA methylation data

**DOI:** 10.1038/s41598-020-65479-y

**Published:** 2020-05-22

**Authors:** Yifan Wang, Ying Wang, Ying Wang, Yongjun Zhang

**Affiliations:** 1 Institute of Cancer and Basic medicine (ICBM), Chinese Academy of Sciences, Zhejiang, China; 2 Ultrasonic Department, Cancer Hospital of the University of Chinese Academy of Sciences, Zhejiang, China; 3 Ultrasonic Department, Zhejiang Cancer Hospital, Zhejiang, China; 4 Department of Basic Medical Science, Zhejiang Chinese Medical University, Hangzhou, China; 5 Department of Gynecological Oncology, Cancer Hospital of the University of Chinese Academy of Sciences, Zhejiang, China; 6 Department of Gynecological Oncology, Zhejiang Cancer Hospital, Zhejiang, China; 7 Department of Integration of Traditional Chinese and Western Medicine, Cancer Hospital of the University of Chinese Academy of Sciences, Zhejiang, China; 8 Department of Integration of Traditional Chinese and Western Medicine, Zhejiang Cancer Hospital, Zhejiang, China

**Keywords:** Non-small-cell lung cancer, Non-small-cell lung cancer

## Abstract

Non–small lung cancer (NSCLC) is a common malignant disease with very poor outcome. Accurate prediction of prognosis can better guide patient risk stratification and treatment decision making, and could optimize the outcome. Utilizing clinical and methylation/expression data in The Cancer Genome Atlas (TCGA), we conducted comprehensive evaluation of early-stage NSCLC to identify a methylation signature for survival prediction. 349 qualified cases of NSCLC with curative surgery were included and further grouped into the training and validation cohorts. We identified 4000 methylation loci with prognostic influence on univariate and multivariate regression analysis in the training cohort. KEGG pathway analysis was conducted to identify the key pathway. Hierarchical clustering and WGCNA co-expression analysis was performed to classify the sample phenotype and molecular subtypes. Hub 5′-C-phosphate-G-3′ (CpG) loci were identified by network analysis and then further applied for the construction of the prognostic signature. The predictive power of the prognostic model was further validated in the validation cohort. Based on clustering analysis, we identified 6 clinical molecular subtypes, which were associated with different clinical characteristics and overall survival; clusters 4 and 6 demonstrated the best and worst outcomes. We identified 17 hub CpG loci, and their weighted combination was used for the establishment of a prognostic model (RiskScore). The RiskScore significantly correlated with post-surgical outcome; patients with a higher RiskScore have worse overall survival in both the training and validation cohorts (*P* < 0.01). We developed a novel methylation signature that can reliably predict prognosis for patients with NSCLC.

## Introduction

With its increasing prevalence, lung cancer has emerged as the main cause of cancer-related deaths in the general population in recent years^1^. Constituting nearly 83% of lung-originated malignancies, non–small cell lung cancer (NSCLC) has a better prognosis than small cell lung cancer, which has consistently dismal outcomes^2^. Despite the favorable prognosis of patients with early-stage NSCLC who undergo curative surgical treatment, up to 40% of these patients would eventually relapse with metastatic disease. The prognosis for these patients remains very poor even with the numerous therapeutic options, including surgery, chemotherapy, target therapy, immunotherapy, etc., for NSCLC^3^. Conventional staging alone is inadequate for prognostic prediction and guidance of treatment decision making. This, there is an urgent need for a novel strategy for risk stratification in NSCLC.

DNA methylation can epigenetically modify genomic expression, and has long been associated with the development and progression of NSCLC as well as several other cancers^4^. It 2005, Schmiemann *et al*. detected the abnormal methylation status of RASSF1A, APC, and p16 (INK4a) in patients with lung cancer; therefore, they proposed the use of methylation detection for early diagnosis of lung cancer^5^. Thereafter, studies on the relationship between lung cancer and gene methylation have increasingly been undertaken. For example, methylation of the MGMT gene promoter was associated with loss or decrease of MGMT expression in lung cancer tissues^6^. Similarly, SHOX2 can be used not only as a marker for early detection of lung cancer, but also as an independent predictor of prognosis for NSCLC^5^. Gene methylation of both SHOX2 and RASSF1A displays high sensitivity not only for the detection of different cancer stages, but also in the identification of different types of lung cancer (e.g., squamous cell carcinoma, small cell lung cancer)^7,8^. Furthermore, the sensitivity of these gene methylation studies increased when the combined methylation of RASSF1A and PCDHGB6 (92%) was evaluated, compared to that of only HOXA9 (80%)^9^. In addition, the combined detection of SHOX2 and PTGER4 gene methylation can improve specificity from 73% to 90%^10^. Overall, the evidence in the literature rationalizes the combination of a series of methylation loci as a prognostic signature for NSCLC. Owing to the recent rapid advances in liquid biopsy, methylation is one of the most popular markers detected in liquid biopsy; it has been shown to be a valid marker for early detection and classification of lung cancer^11–14^.

The Cancer Genome Atlas (TCGA) – a landmark cancer genomics program and the largest database of cancer – comprises molecular characterizations of over 20,000 primary cancer as well as matched normal samples spanning 33 cancer types. The TCGA program has generated, analyzed, and made available genomic sequence, expression, methylation as well as corresponding survival data, which makes it a perfect source for the identification of novel genomic/epigenomic markers with prognostic significance^15^. On the basis of the TCGA methylation spectrum of NSCLC, we sought to develop a prognostic model that integrates the most important methylation loci with their prognostic significance. By this study, we aimed to gain more insights into NSCLC survival prediction.

## Materials and Methods

### Data collection

We included all cases of non–small cell lung cancer with epigenomic and genomic data as well as clinical data available on TCGA (The Cancer Genome Atlas). We collected clinical information on age, sex, race, history, type of diagnosis, and tumor stage of NSCLC from the TCGA database, on the website of National Cancer Institute (https://cancergenome.nih.gov/). We used the TCGA GDC API to download the latest clinical follow-up information, 450k methylation data, and the TCGA RNA-Sequence data of NSCLC. All data were collected on November 13, 2018. Subsequently, we collected follow-up information of 504 cases, RNA-Seq data of 551 cases, and Illumina Infinium HumanMethylation450 data of 415 cases.

### Data preprocessing

For further analysis, we included a total of 349 cases with available clinical, methylation, and mRNA sequence data that had follow-up time of more than 30 days. Samples of 5′-C-phosphate-G-3′ (CpG) sites with NA (not available) value of more than 70% were removed; meanwhile, we removed the CpG sites with cross-reactivity on the basis of the discovery of cross-reactive probes and polymorphic CpGs in the Illumina Infinium HumanMethylation450 microarray, as reported previously^16^. The KNN method in R package (imputeR: A General Multivariate Imputation Framework) was used to impute the deletion value to the methylation spectrum, and to further exclude unstable genome methylation sites – the CpGs and single-nucleotide sites on the sex chromosome. Finally, we obtained 208,022 methylation sites.

### Sample grouping

We randomly divided 349 samples into training (n = 174) and validation (n = 175) sets that were matched for age distribution, clinical staging, follow-up time, and mortality rate (Table 1). None of the included patients had received any adjuvant chemotherapy or radiotherapy. We carried out identification of prognostic methylation loci, hierarchical analysis, pathway analysis, weighted correlation network analysis (WGCNA) co-expression analysis, and construction of a prognostic model in the training cohort. In the validation cohort, we undertook validation of the predictive power of the prognostic model.

**Table 1:** Basic clinical information of Training cohort and Validation cohort.

		Training cohort	Validation cohort	P value (Chi-square)
Gender	Male	129	129	0.9282
	Female	45	46	
Age (year)	40–49	7	7	0.7341
	50–59	24	26	
	60–69	53	63	
	70–79	79	66	
	80–84	8	11	
	Not Available	3	2	
T	T1	18	13	0.5684
	T1a	9	11	
	T1b	17	18	
	T2	38	46	
	T2a	43	35	
	T2b	17	15	
	T3	29	28	
	T4	3	9	
N	N0	112	111	0.4453
	N1	44	50	
	N2	17	11	
	NX	1	3	
M	M0	138	135	0.5505
	M1	0	1	
	M1a	0	1	
	M1b	0	1	
	MX	36	37	
smoking	smoked	156	153	0.5138
	Not Available	18	22	

### Statistical analysis

Most of the statistical analysis was undertaken on SPSS software (version 19.0, IBM Corp., Armonk, NY, USA). Specific analysis was carried out by R language 3.1.4 (http://www. r-project.org) in Rpackage.

#### Identification of prognostically significant methylation loci

A univariate Cox proportional hazard regression model was developed on the training set, considering all the methylation sites of the whole genome as well as clinicopathological parameters, such as age, gender, and T, N, and clinical stages. Further multivariate Cox proportional hazard regression will be carried out on variables with significant influence on overall survival in univariate analysis. All analyses were implemented by the coxph function in Rpackage. *P*-value less than 0.05 was considered indicative of statistical significance.

#### Hierarchical clustering

**Figure 1:**
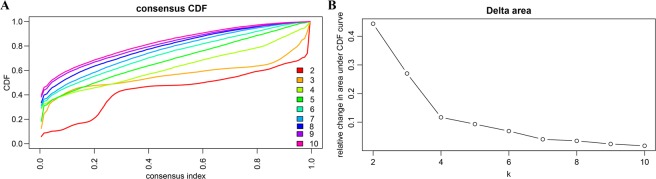
(**A**) Curve of cumulative distribution function (CDF), (**B**) CDF delta area curve of consensus clustering, with the x axis representing the category k, and the y axis denoting the relative change in area under CDF curve of category k when compared with category k − 1.

We conducted unsupervised hierarchical clustering for the methylation levels of the methylation loci that were found to be independent prognostic parameters on multivariate analysis. The similarity distance between samples was used to calculate the Euclidean distance. The optimal clustering number was determined by the cumulative distribution function (CDF; Fig. 1A). A double sampling plan was adopted, with 80% of samples sampled each time and repeated a 100 times. Figure 1 shows that the stability of the result can be achieved when the number of clusters (K) is up to 6, and this was selected as the cluster number for further analysis. Clustering analysis was carried out with the Consensus Cluster Plus of R software package (*P*-value <0.05 was considered statistically significant).

#### KEGG pathway analysis

We included all prognostically significant methylation loci to the Kyoto Encyclopedia of Genes and Genomes (KEGG) function enrichment analysis, which was conducted by the clusterProfiler package in R software. KEGG pathways with *P*-values <0.05 were identified as statistically significant.

#### WGCNA co-expression analysis

**Figure 2:**
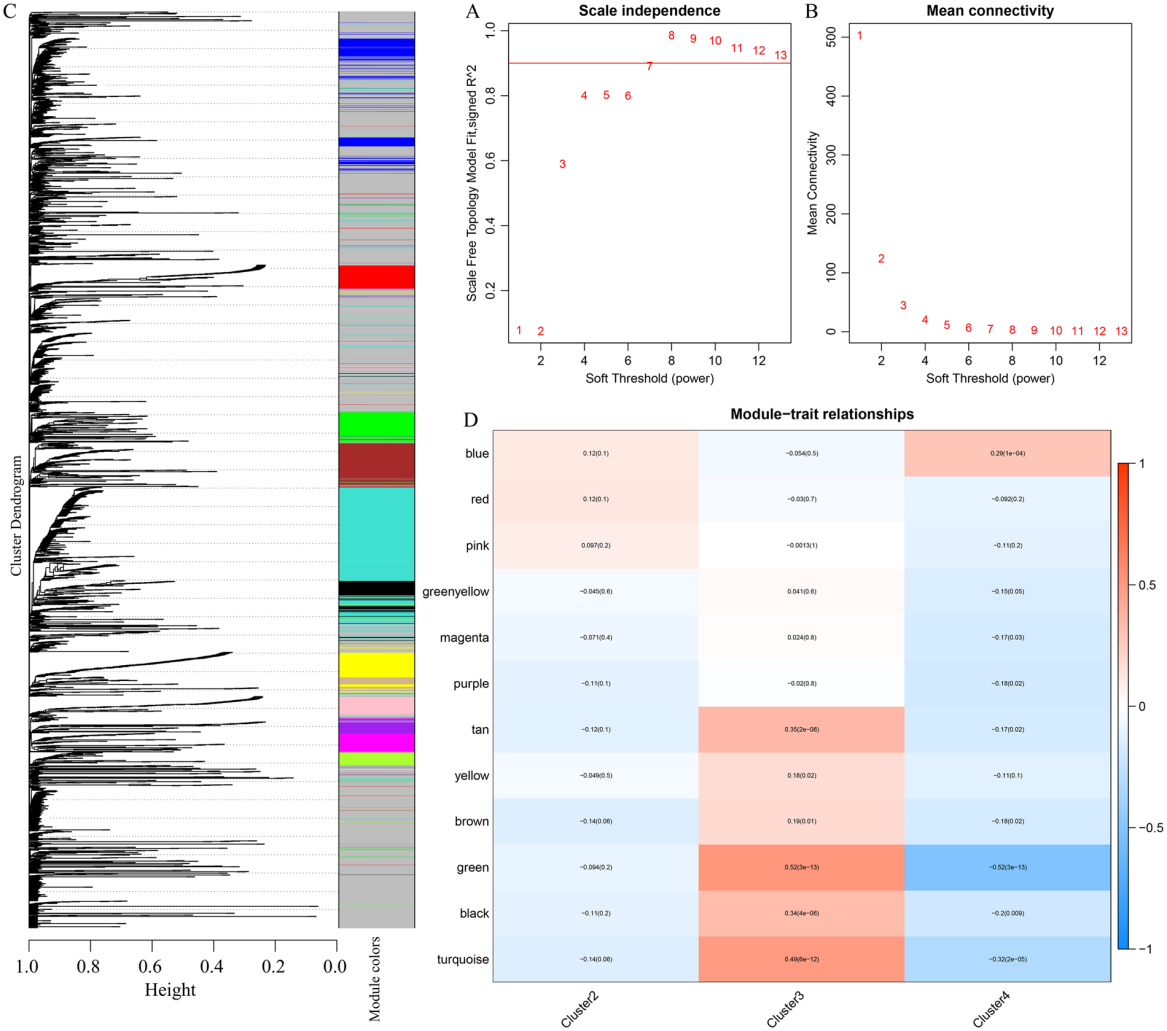
(**A**,**B**) Network topology analysis for different soft-thresholding powers; (**C**) gene dendrogram and module colors; and (**D**) correlation between gene module and characteristic clusters.

We conducted the WGCNA co-expression analysis in the R software package WGCNA, and applied the WGCNA co-expression algorithm to explore the co-expression of CpG sites among all prognostically significant methylation loci. Then, we calculated the distance between each CpG site using the Pearson correlation coefficient, and constructed a weighted co-expression network. The analysis showed the co-expression network conforms to a scale-free network – that is, the log log(k) of the node with connection degree k negatively correlates with the log log(P(k)) of the probability of the node; the correlation coefficient is greater than 0.8. To ensure the network is scale-free, we selected beta = 7 (Fig. 2A,B). First, the expression matrix was transformed into an adjacency matrix, and, subsequently, into a topological matrix (TOM). Based on the TOM, we used the average-linkage hierarchical clustering method to cluster genes; thereafter, we followed the standard of mixed dynamic shear tree, and set the minimum number of genes in each long non-coding RNA (lncRNA) network module to 30. After we determined the gene modules by a dynamic shearing method, the eigengenes of each module were calculated in turn. Then, we undertook cluster analysis on the modules; the modules with proximity were merged into new modules (height = 0.25, deepSplit = 2, and minModuleSize = 30).

## Results

### Identification of methylation sites with prognostic influence

To identify the methylation loci or clinical parameters associated with overall survival, we conducted a univariate cox proportional hazard regression analysis of the training cohort. We obtained 9201 loci with significant prognostic impact (*P* < 0.05); the top 20 of these loci are shown in Table 2. Among the clinical parameters, including age, gender, T stage, N stage, and clinical stage, only the T stage and clinical stage were found to be significantly associated with prognosis (*P* = 0.0002197 and 0.005511, respectively).

**Table 2:** Top 20 methylation loci with significant prognostic influence.

CpGs	*P*-value	HR	Low 95%CI	High 95%CI
cg15804782	4.86E − 07	8.59E + 14	1.31E + 09	5.64E + 20
cg05767633	7.58E − 07	8.45E + 18	2.67E + 11	2.67E + 26
cg09038676	8.63E − 07	3166428	8152.608	1.23E + 09
cg01097611	1.12E − 06	1.44E + 10	1177287	1.77E + 14
cg21348997	1.55E − 06	1.52E + 12	16342722	1.42E + 17
cg04216397	2.59E − 06	1.27E + 20	5.29E + 11	3.07E + 28
cg06894812	4.25E − 06	2.23E + 12	12164167	4.08E + 17
cg05324014	4.45E − 06	6.32E-07	1.42E − 09	0.000281
cg27628312	7.92E − 06	3.82E + 18	2.69E + 10	5.42E + 26
cg09110402	9.79E − 06	85.08886	11.87047	609.9263
cg02726924	1.12E − 05	3.09E + 19	6.19E + 10	1.54E + 28
cg22294241	2.43E − 05	654985	1305.684	3.29E + 08
cg26820911	2.46E − 05	1.88E + 11	1086987	3.26E + 16
cg00191629	2.59E − 05	3545.787	78.70704	159739.3
cg06742044	2.86E − 05	2.62E + 10	345090.2	1.99E + 15
cg17074000	3.11E − 05	82.28059	10.32936	655.4223
cg10070969	3.29E − 05	108264.7	455.2979	25744130
cg03862040	3.48E − 05	7.86E + 31	6.22E + 16	9.94E + 46
cg02442412	3.51E − 05	18951994	6766.073	5.31E + 10
cg26944011	3.68E − 05	7.62E + 09	154362.6	3.76E + 14

Thus, we further applied the T stage, N stage, and pre-identified 9201 methylation loci in a multivariate Cox proportional risk regression analysis to screen out independent prognostic markers. 4000 methylation sites were found to be independently correlated with overall survival in NSCLC.

### Hierarchical cluster analysis of prognosis-associated loci

**Figure 3:**
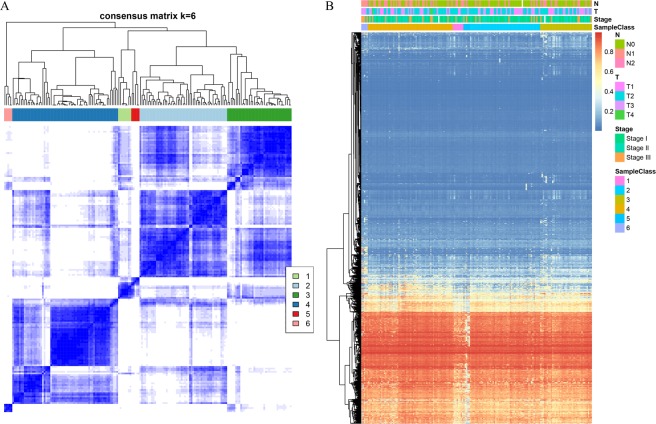
(**A**) Clustering heatmap in the case of consensus k = 6; (**B**) methylation heatmap of 4000 methylation loci in the training cohort.

The unsupervised hierarchical clustering of the 4000 prognosis-associated loci separated all 174 samples in the training cohort into six categories (Fig. 3A). The heatmap analysis (Fig. 3B) showed most of the methylation sites manifested low abundance. However, samples of the 6 categories manifested different methylation patterns (Fig. 3B).

**Figure 4:**
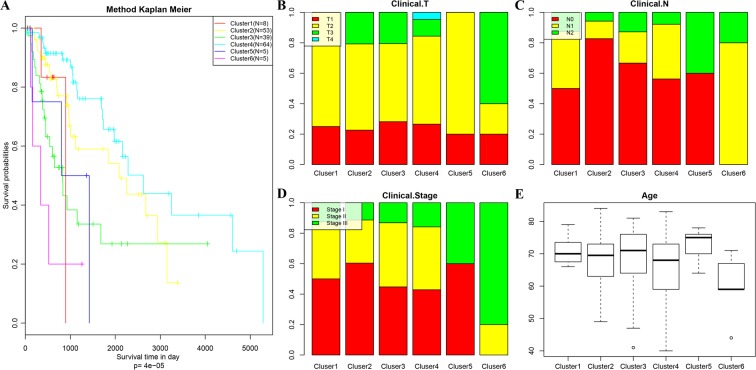
(**A**) Prognostic differences among 6 models; (**B**) proportion of different T stages in 6 models; (**C**) The proportion of different N stages in 6 models; (**D**) The proportion of different clinical stages in 6 models; and E. age distribution in 6 models.

Furthermore, we analyzed clinicopathological features of the 6 molecular subtypes in regard to distribution of: T, N, and clinical stages; age; and difference in overall survival. We observed significant prognostic differences among the 6 molecular subtypes (p = 4e-5; Fig. 4A); the best prognosis was achieved in Cluster 4, and the worst in Cluster 6. The clinicopathological parameters had different distribution patterns among the 6 clusters. In general, patients in Cluster 6 tended to have a later clinical stage, larger tumor size, and more lymph node metastases; however, they were younger (details in Fig. 4B–E). These results validate the use of molecular subtypes to classify patients who have different outcomes in addition to their clinical features.

### Pathway analysis of prognosis-associated loci

**Figure 5:**
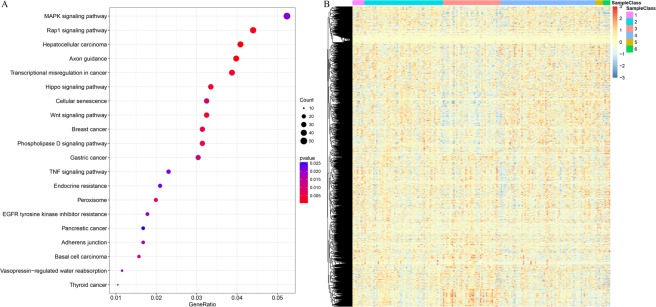
(**A**) KEGG pathway enrichment analysis of 4000 methylation with prognostic significance; (**B**) expression profile of 2747 genes corresponding to 4000 methylation with prognostic significance.

All the 4000 methylation loci that manifested prognostic influence were detected on annotation and pathway analysis. All of these 4000 methylation loci were mapped to 3482 genes. As demonstrated on the KEGG function enrichment analysis, the 3482 genes were mainly enriched in multiple signaling and cancer pathways, such as the MAPK signaling pathway, VEGF signaling pathway, central carbon metabolism in cancer, transcriptional dysregulation in cancer, and so on; these are known to be closely related to tumorigenesis and development (Fig. 5A).

To further explore the expression profile of the 3482 genes identified in our study, we obtained the mRNA sequence profile of these genes from the TCGA database for training cohort. The mRNA expression profile was available for only 2747 genes, and these were applied in the heatmap analysis for 174 samples in the training cohort. As shown in Fig. 5B, samples of different methylation clusters demonstrated similar mRNA expression patterns, thereby suggesting that the DNA methylation levels and gene expression of these genes are consistent.

### Classification of molecular subtypes

From the WGCNA co-expression analysis, we obtained 13 modules (Fig. 2C). All methylation loci that could not be aggregated into other modules were assembled as the gray module. As shown in Table 3, 1833 CpG were allocated to 12 modules. As clusters 1, 5, and 6 contained few samples, we only selected clusters 2, 3, and 4 as the three main categories of the samples, and their correlation with each module was analyzed by the Pearson correlation analysis. Cluster3 positively correlated with most modules, whereas Cluster4 demonstrated a negative correlation with the majority of modules (Fig. 2D).

**Table 3:** Number of CpG loci in each module.

Module	CpG count
Brown	187
Green	144
Greenish yellow	62
Magenta	93
Pink	99
Purple	71
Red	117
Tan	35
Turquoise	570
Yellow	145
Black	111
Blue	199

### Hub CpG loci screening

**Figure 6:**
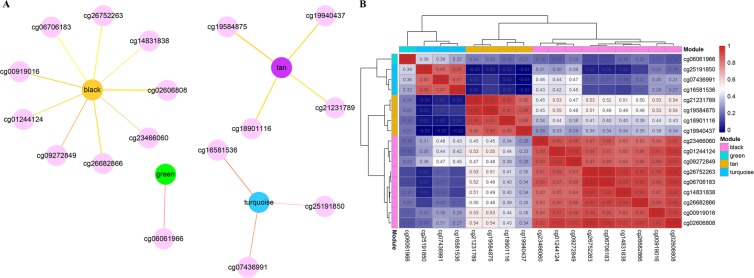
(**A**) Association between hub CpG loci and different modules; (**B**) association between hub CpG loci and characteristic clusters.

The blue, tan, green, black, and turquoise modules showed significant correlation with both Cluster3 and Cluster4, we selected the methylation sites in these modules and calculated their correlation with the corresponding modules (MM) as well as with the Cluster3 phenotype (GS). We identified the hub CpG loci by MM > 0.9 and GS > 0.2 (Fig. 6A). There were 17 CpG loci in total, and most were in the black module. Table 4 shows detailed annotation information of the 17 CpG loci. These CpG sites were annotated on 16 genes, and 14 CpG sites were located on the gene promoter CpG island. Furthermore, we analyzed the methylation correlation among the 17 CpG loci by hierarchical clustering analysis. In Fig. 6B, we show where the correlation between the genes in each module is the highest and lowest.

**Table 4:** Annotation information of the 17 hub CpG loci.

CpG	Chrom	Start	End	GeneSymbol	Feature_Type	MM	GS	Module
cg02606808	chr5	72107675	72107676	MAP1B	Island	0.947354	0.24915	black
cg19940437	chr14	89954878	89954879	EFCAB11	S_Shore	0.934014	0.275199	tan
cg18901116	chr10	71397101	71397102	CDH23	Island	0.939698	0.259864	tan
cg19940437	chr14	89954878	89954879	TDP1	S_Shore	0.934014	0.275199	tan
cg00919016	chr7	1.39E + 08	1.39E + 08	KLRG2	Island	0.930312	0.289498	black
cg25191850	chr1	2.34E + 08	2.34E + 08	KCNK1	Island	0.900188	0.49867	turquoise
cg14831838	chr2	2.19E + 08	2.19E + 08	CDK5R2	Island	0.909691	0.264816	black
cg26682866	chr2	2.19E + 08	2.19E + 08	CDK5R2	Island	0.935384	0.262505	black
cg19584875	chr14	90061869	90061870	KCNK13	Island	0.942315	0.279599	tan
cg21231789	chr14	90061855	90061856	KCNK13	Island	0.932377	0.254928	tan
cg16581536	chr14	37595644	37595645	TTC6	Island	0.916525	0.46462	turquoise
cg16581536	chr14	37595644	37595645	FOXA1	Island	0.916525	0.46462	turquoise
cg06706183	chr6	53545058	53545059	GCLC	Island	0.923999	0.204057	black
cg26752263	chr6	53545055	53545056	GCLC	Island	0.930967	0.217814	black
cg19940437	chr14	89954878	89954879	RP11-33N16.3	S_Shore	0.934014	0.275199	tan
cg23466060	chr4	13544858	13544859	NKX3-2	Island	0.908867	0.326585	black
cg06061966	chr11	46345093	46345094	DGKZ	N_Shore	0.905048	0.551368	green
cg01244124	chr15	70763776	70763777	UACA	Island	0.925507	0.263363	black
cg09272849	chr15	70763496	70763497	UACA	Island	0.917523	0.35689	black
cg07436991	chr20	11890663	11890664	BTBD3	N_Shore	0.911026	0.45042	turquoise

### Establishment and validation of prognostic model

From the 17 hub CpG loci, we constructed a prognostic signature that was a weighted combination of these prognostic markers. We selected the methylation spectra of these 17 CpG sites, and determined the modification abundance of each CpG site in each sample. We used multifactor regression to analyze the 17 CpG loci and established a RiskScore model: According to the modified abundance of the 4000 CpG sites we obtained, we weighted the correlation coefficients of genes as the elements in the co-expression matrix by using the principles of the WGCNA co-expression algorithm. The weight-selection criterion was to used for the subduction of the connection between the genes contained in each gene network without a scale network distribution. Thus, the logarithm (log (I)) of the number of connected nodes is negatively correlated with the log (p(I)) of the probability of the occurrence of this node. Then, we determined the value of the weighted coefficient, and a risk score model was established on the basis of multivariate regression analysis with the formula:

$$\begin{array}{c}RiskScore=\\ 6.81\times cg25191850+17.73\times cg21231789+\,0.5\times cg14831838+\\ 6.92\times cg00919016-10.15\times cg07436991+\,2.4\times cg26682866-\\ 14.78\times cg01244124+3.07\times cg19584875-3.09\times cg09272849+\\ 4.29\times cg02606808-9.02\times cg18901116+0.81\times cg06061966-\\ 40.77\times cg26752263-2.33\times cg23466060+\,3.91\times cg16581536-\\ 9.42\times cg19940437+71.2\times cg06706183\end{array}$$

**Figure 7:**
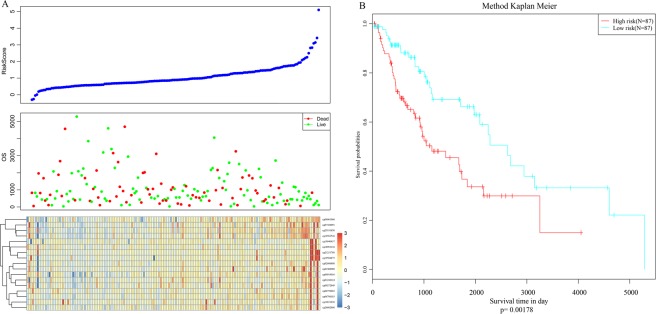
(**A**) Correlation of RiskScore with methylation pattern and overall survival in the training cohort; (**B**) Kaplan–Meier survival analysis of patients with high RiskScore vs low RiskScore in the training cohort.

For each score, we calculated the risk score for each sample, and observed the expression patterns of CpG corresponding to different risk scores and their relationship with the overall survival. Together with the gradual increase of the RiskScore, the methylation level of the samples at the 17 CpG sites increased gradually, whereas the overall survival showed a decreasing trend (Fig. 7A). The median value of the RiskScore was used as the cutoff value to classify the samples into high-risk (RiskScore> median) and low-risk (RiskScore <median) groups. The high-risk group had a significantly worse overall survival than the low-risk group (*P* = 0.00178; Fig. 7B).

**Figure 8:**
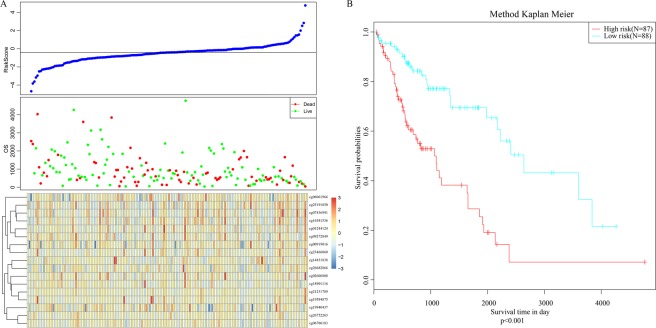
(**A**) Correlation of RiskScore with the methylation pattern and overall survival in the validation cohort; (**B**) Kaplan–Meier survival analysis of patient with high RiskScore vs low RiskScore in the validation cohort.

We applied the same RiskScore model to the validation cohort and evaluated its predictive power with regard to prognosis. The correlation of the RiskScore with methylation pattern and overall survival in the validation cohort was similar to that in the training cohort (Fig. 8A). Moreover, patients with a RiskScore larger than the median value had significantly worse prognosis than those with a lower RiskScore (*P* < 0.001; Fig. 8B). We inferred that the prognostic model constructed by the methylation spectrum of these 17 CpG sites can reliably predict prognosis for patients with NSCLC.

## Discussion

Despite considerable research efforts into NSCLC in the past decade, there is no significant improvement in the overall survival of patients with NSCLC, especially those with early-stage NSCLC. The identification of high-risk early-stage NSCLC and early implementation of an enhanced therapeutic regimen is the key to improve the cure rate for NSCLC. Risk-stratification tools can better guide clinical decision making for early-stage lung cancer that is at high risk of relapse and, in these cases, multimodality treatment should be considered. The conventional staging and grading systems cannot fully identify patients with NSCLC who are at high risk of relapse, especially early-stage patients who have a risk of recurrence after curative surgery.

With the clinical and genomic/epigenomic data we extracted from the TCGA database of patients with NSCLC who underwent curative surgery, we sought to establish a risk-stratification model on the basis of methylation markers. Subsequently, we identified 17 methylation loci with significant prognostic influence and used them to construct the prognostic model; the prediction power of this model was confirmed in the validation dataset. The results proved the methylation-based prognostic signature was a valid marker for risk stratification in early-stage NSCLC.

The rapid development of the high-throughput genomic/epigenomic detection technology facilitated further molecular insights into subgroup characteristics, from the perspective of gene mutation, gene expression, DNA methylation, and protein expression profiles, of patients with NSCLC. Genome-wide data have made it feasible to screen for core prognostic molecules, and the combination of these conveys stronger predictive power in terms of diagnosis or prognosis when compared with the predictive power of a single marker. Several of the previous studies have focus on mRNA expression data to develop prognostic signatures for all types of malignant diseases, including lung cancer. Microarray and RNA sequence analyses have produced tons of tumor RNA expression signatures that are associated with clinical outcomes in NSCLC^17–23^. However, none of these prognostic expression signatures have been applied to clinical practice because of their uncertain performance on clinical samples. Furthermore, some studies were devoted to the excavation of prognostic markers from the perspective of microRNA or lncRNA profile, and a few prognostic signatures were proposed^24–26^; however, their clinical performance remains to be evaluated.

DNA methylation is another potential biomarker known to convey diagnostic and prognostic significance in many types of cancer^27^. DNA methylation is an epigenetic mechanism that modifies a cytosine base through the addition of a methyl group at the CpG nucleotide residues (4). Vertebrate CpG islands are short, interspersed DNA sequences that are rich in guanine and cytosine (GC) and are predominantly non-methylated^28,29^. The development of lung cancer has been associated with the exposure to hazardous environmental substances through respiration, which is considered a common cause of alteration in genome methylation^30,31^. The methylation status of specific genes has been found to be of diagnostic and prognostic value in lung cancer^32,33^. Genomic-wide methylation analysis has enabled the screening of methylation loci that have prognostic influence. Methylation-based diagnostic or prognostic signatures have been proposed for many cancers, including breast cancer, melanoma, colon cancer, hepatocellular cancer, and so on, and has shown promising predictive power^34–39^. A previous study proved DNA methylation would be a better biomarker for diagnosis and prognosis because of its predictive stability when compared with gene and miRNA expression profiles^40^. A 4-gene methylation signature was recently proposed to predict the outcome of early-stage lung adenocarcinoma^41^. Nonetheless, studies on a methylation-based prediction model for NSCLC are scarce.

In this study, we screened the hub methylation loci and established a prognostic model on the basis of the loci; this model was confirmed to be reliable in outcome prediction for early-stage lung cancer in the validation cohort. Our findings support the feasibility of methylation signature in risk stratification of patients with early-stage NSCLC. We identified 17 hub methylation loci that correspond to 13 genes; some of these have been previously shown to be associated with tumorigenesis in lung cancer. For example, the epigenetic repression of MAP1B was associated with the development of lung cancer in patients with chronic obstructive pulmonary disease^42^. Cdh23 functions as a suppressor of cell migration, and its deletion can lead to progression of lung cancer^43^. GCLC is another tumor suppressor gene that induces synthetic lethality of cancer cells; GCLC deletion is associated with lung cancer development^44^. Moreover, CDK5R2/p39 increased the invasiveness of lung cancer by impairing cell adhesion and promoting epithelial-to-mesenchymal transition^45^. FOXA1 promoted lung cancer development as a suppressor of the tumor immune microenvironment, which facilitates immune evasion of cancer cells^46^. The consistency between the results of our study and the previous studies further confirms the reliability of our current findings, and rationalizes the use of the methylation spectrum for risk stratification in NSCLC. Also, further analysis of the novel markers that identified in our study may generate more insights into the mechanism of lung cancer etiopathogenesis, or hopefully lead to the identification of new therapeutic targets.

In comparison with the methylation levels of selected genes, the RiskScore model integrating all the prognosis-related gene loci can yield more precise results and facilitate better risk stratification. The methylation based scoring system can be incorporated in the clinical practice for risk stratification of patients with early-stage lung cancer who have undergone surgical treatment. RiskScore can help predict the risk of recurrence and guide decision-making with respect to application of adjuvant therapy. In addition, this risk scoring model can also be applied to patients with advanced lung cancer for predicting long-term outcomes and to determine the best therapeutic choice. As tissue samples may not be available from patients who do not undergo surgery, circulating tumor DNA may serve as a promising substitute for methylation detection. As our RiskScore model is based on tissue, further study is required to validate its application on liquid samples like plasma, sputum, or bronchoalveolar lavage fluid. Applying RiskScore with liquid biopsies can help in dynamic monitoring of the therapeutic effect and disease progression.

Our study sheds light on improving the clinical management of early-stage NSCLC by enhancing risk stratification through the methylation profile. However, this study has some limitations that should be clearly addressed. First, we could not test our model in the setting of predicting the risk of recurrence, as data pertaining to progression-free survival data are not available in the TCGA database. As indicated in ESMO guidelines, the risk of recurrence ranges from 6%–10% per person per year, but decreases thereafter to 2%. After 5 years, the recurrence is virtually absent. This implies that long overall survival may be equivalent to lack of recurrence. Our results based on overall survival may reflect the risk of recurrence to some extent. However, further studies based on recurrence data are still required. Also, the methylation profile used in our study was derived from fresh frozen surgical samples; it remains unknown whether our results can be replicated in formalin-fixed and paraffin-embedded (FFPE) tissue samples or on clinical samples. The reliability of our findings should be further verified in the clinical settings. Furthermore, the prognostic model established in our study is a combination of the weighted level of certain methylation loci. The weight coefficient may change when data are produced by another analysis platform or how the quantifying methylation level is altered; this could limit the widespread application of the established RiskScore model. The model is yet to be simplified to be feasible for clinical application. Lastly, the methylation signature alone may not potentiate its value in prognostic prediction. Further effort is required to integrate the methylation signature with other prognostic markers such as clinicopathological parameters, genomic mutation, or gene expression profile to maximize the predictive power of the model.

In conclusion, we identified a prognostic methylated NSCLC classifier based on the TCGA methylation spectrum. This classifier can efficiently identify patients of early-stage NSCLC with high risk of recurrence, wherein multimodality treatment should be considered. This model can guide clinicians in the selection of the most appropriate therapeutic for different individual, and thus optimize the clinical outcome of patients with NSCLC.

## Data Availability

The datasets generated and analyzed during the current study are available from the corresponding author on reasonable request.
